# Understanding the Relationship between Distress Behaviour and Health Status of People with Autism Spectrum Disorder

**DOI:** 10.3390/healthcare11111565

**Published:** 2023-05-26

**Authors:** Antonio Koceski, Callum J. Smith, Yasir Ahmed Syed, Vladimir Trajkovski

**Affiliations:** 1 Primary School “Blaze Koneski”, 7500 Prilep, North Macedonia; 2 Neuroscience and Mental Health Innovation Institute, Hadyn Ellis Building, Cardiff CF24 4HQ, UK; 3 School of Bioscience, Cardiff University, The Sir Martin Evans Building, Museum Ave., Cardiff CF10 3AX, UK; 4 Macedonian Scientific Society for Autism, Institute of Special Education and Institute of Special Education and Rehabilitation, Faculty of Philosophy, University “Ss. Cyril and Methodius”, 1000 Skopje, North Macedonia

**Keywords:** Autism Spectrum Disorder, challenging behaviour, social deficits, diet, health status

## Abstract

Autism Spectrum Disorder (ASD) is associated with complex distress and challenging behaviours that have a negative impact on the everyday life of those with ASD, as well as their parents and carers. These challenging behaviours include negative emotional behaviours, motor behaviours, and changes in routines. Even though challenging behaviours occur in most subjects with ASD, the cause of these largely remains unknown. It has been implicated that these challenging behaviours are associated with a change in the health of those with ASD. More research needs to be conducted that can establish a direct association. Towards this goal, the present study aimed to explore whether health status had an impact on the distressing behaviour in the subjects diagnosed with ASD. We analysed the response from the parents/carers in a Macedonian population of those with ASD, to determine which challenging behaviours were most likely to be observed during a change in health. Based on a scoring system, the manifestation of challenging behaviour was evaluated and compared with the changes in health. Changes in appetite or dietary preferences, irritability and low mood, and loss of previously acquired skills had the greatest association with a change in health. These findings provide early insight into types of challenging behaviours that are directly associated with a change in health. Our results demonstrate a relationship between health status and challenging behaviour in the subject with autism, suggesting that caregivers may need to consider this when choosing strategies for managing challenging behaviour.

## 1. Introduction

Autism Spectrum Disorder (ASD) is associated with complex challenging behaviours that interfere with the everyday life of those with ASD and those who care for them [1]. Challenging behaviours can be defined as a type of behaviour that is not socially acceptable, affects their education, and physically harms the individual and others. These behaviours include negative emotional behaviours, motor behaviour, vocalisations, changes in routines, and habits. Research shows that up to 94.3% of the children and adolescents with ASD demonstrate at least one challenging behaviour [2] regardless of their gender [3]. Challenging behaviour in ASD could be due to an intentional effort to communicate and or related to sensory stimming. It can also be in response to the onset of fear, distress, aggression, muscle weakness, and digestive discomfort and need to be interpreted within the context of the patient’s environment, communication skills, and other co-morbidities [4,5]. Some of the pathobiological processes that are associated with ASD, might also result in challenging behaviours, which could be outside subject’s control [6]. An additional factor in the incidence of challenging behaviours is the severity of symptomatology, IQ, and functional status of the individual with ASD [2,4].

It has been shown that changes in the health of the individual with ASD are associated with the manifestation of challenging behaviours. According to Buie et al. (2010), challenging behaviours such as irritability and low mood, anxiety, tantrums, self-harm, loss of previously acquired skills, motor behaviours, and aggression can be due to changes in health status [7]. These changes in health may include otitis media, otitis externa, pharyngitis, sinusitis, epilepsy, dental abscess, constipation, urinary tract infection, fracture, headache, esophagitis, gastritis, colitis, allergies, and others [8,9,10]. It has been suggested that those with low-functioning ASD often have more severe health problems, and this could lead to more frequent and serious manifestations of challenging behaviours, such as anger management issues, aggression, environmental destruction, socially inappropriate behaviour, and self-harm [11]. Furthermore, it has been shown that there is an association between the manifestation of challenging behaviours and sleep disorders such as obstructive sleep apnoea [12,13]. Eating disorders [14], aggressive behaviour [15], and post-traumatic stress disorder [16] have also been shown to be associated with the manifestation of challenging behaviours.

Given that some changes in health can be obvious to those who care for individuals with ASD, it can be relatively easy to determine which health changes are associated with the manifestations of challenging behaviours. Although, most changes in health are not obvious to carers of those with ASD. For example, children with ASD can have endocrinological and metabolic abnormalities that are not detectable without the right clinical knowledge and diagnosis. Furthermore, communication is difficult for those with ASD and therefore, they are not able to verbally communicate their symptoms to carers. Therefore, challenging behaviours can be seen as a method of communication and an attempt to convey a problem with their health. Without proper intervention, these behaviours tend to continue and may even worsen, to become a chronic behavioural problem [17,18]. This will lead to an increase in careering responsibilities and burden on the families of the subjects with ASD [7]. If the cause of the challenging behaviour is a change in the health of the individual, then the correct medical intervention to treat the health condition is likely to result in the resolution of the challenging behaviours.

By determining which challenging behaviours are frequently present during a change in health, then these behaviours could be used as an indication of a medical problem. This is likely to prompt parents/carers to seek medical attention to be more vigilant of other signs of illness. As very few studies have documented the challenging behaviour that is observed during a change in the health of those with ASD [7,9], we aimed to address this knowledge gap. We conducted a retrospective cohort study in which questionnaires were sent to carers/parents of those with ASD in a Macedonian population. Participants provided information on behaviours that were observed during a change in the health of the individual with ASD. We sought to determine which challenging behaviours were frequently observed during a change in health and determine if there is a correlation between these behaviours.

## 2. Methods

### 2.1. Research Setting and Design

The study was approved by the faculty of Philosophy in Skopje Institutional Review Board approval (10-487/5). This study includes a total of 72 respondents residing in North Macedonia with diagnosed ASD according to the DSM-5 criteria. The age of the respondents ranges from 3 to 24 years. The number of particpants <10 years was 29, between 11–20 years was 42, and >20 years was 1. The research was conducted in the period from January to July 2019 in most areas of North Macedonia (Skopje, Bitola, Prilep, Veles, Tetovo, Ohrid, Struga, Stip, Kicevo, Kavadarci, and the village of Krivogashtani). The participants of the study were sourced through the Macedonian Scientific Society for Autism, which provided direct contact with the institutions for access to the target groups, which was conducted through a telephone conversation with a parent/guardian. The institutions in which the research was conducted were the Special Schools “Idnina” and “Zlatan Sremac” in Skopje, Daily Center for Autism in Skopje, non-governmental organisation “Open the windows” in Skopje, Center for Psycho-Physical Health, “Gaspar” in Skopje, Primary School “Kocho Racin” in Bitola, “Institute for Rehabilitation of Hearing, Speech and Voice” in Bitola, Public Institution Kindergarten “Our Future” in Prilep. In other cities, the data were taken by telephone. The respondents have made a diagnosis in the appropriate centres and institutions in the territory of North Macedonia.

### 2.2. Design and Measures

Information on challenging behaviours was collected through a survey completed by the parents/guardians of the individuals with ASD using the “Challenging Behaviour Questionnaire” which aims to determine how often challenging behaviours occur, whether the presence of these challenging behaviours was closely related to changes in health status and whether the challenging behaviours continued to persist even after the health status had improved. The questionnaire consists of a list of challenging behaviours was based on a previously published study [7]. The questionaire asked the participants asked to identify the different challenging behaviours observed in the individual with ASD and whether the reason for the onset of this behaviour has an association with changes in their health status, with participants answering yes or no to each behaviour. A numerical scale from 1 to 5 was also included for each behaviour to allow the participants to score the reliability and severity of the behaviour.

### 2.3. Data Analysis

The data from this research were graphically processed and tabulated in the statistical software SSPS V.26.0. Statistical analysis was performed using the One-Sample *t*-Test as no comparison had been made across variables. Correlation analysis was performed to see the correlation between the results obtained from the subjects. A 95% confidence interval, mean score (μ), and Pearson’s correlation coefficient (r) across challenging behaviour types were used. The statistical significance was determined at the level of *p* < 0.05.

## 3. Results

### 3.1. Demographic Characteristics of the Participants

**Figure 1:**
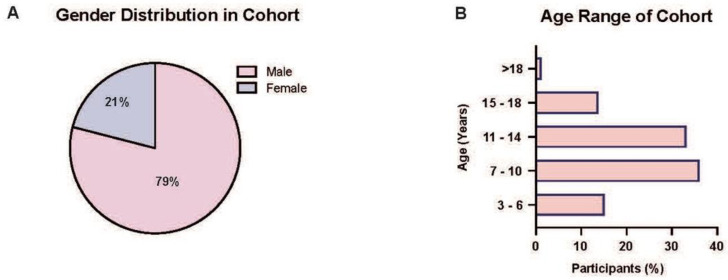
Demographic status of cohort. (**A**) Pie chart showing the gender distribution in the cohort. 79% (*n* = 57) male, and 21% (*n* = 15) female. (**B**) Bar graph showing the distribution of the age across cohort. 15.28% (*n* = 11) 3–6 years, 36.11% (*n* = 26) 7–10 years, 33.33% (*n* = 24) 11–14 years, 13.89% (*n* = 10) 15–18 years, and 1.39% (*n* = 1) >18 years).

A total of 72 respondents were included in this analysis. Table 1 shows the respondent demographic data. Of these, a total of 15 respondents (21%) were female and 57 respondents (79%) male, with a female-to-male ratio of 1:3.8 (Figure 1A and Table 2). The age range of respondents was 3–24 years (Figure 1B); the average height, weight, and waist circumference of the cohort being 142.60 ± 26.06 cm, 46.26 ± 20.15 kg, 63.46 ± 20.13 cm, respectively (Table 1).

#### 3.1.1. Challenging Behaviours Observed during a Change in Health Status

Challenging behaviours were grouped into associated behaviours such as negative emotional behaviours, changes in routine and skill, and motor and vocal behaviour. Types of challenging behaviour (Table 2), the frequency in which the challenging behaviour occurred, and the correlation between behaviours was investigated using the participant data.

#### 3.1.2. Negative Emotional Behaviours

**Figure 2:**
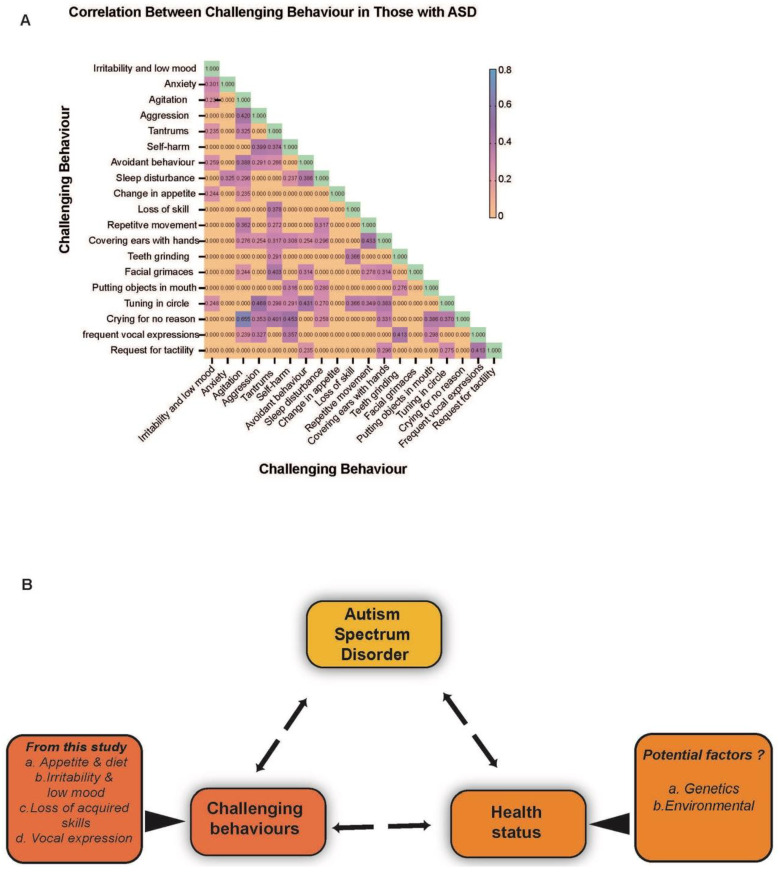
Correlation between challenging behaviors in those with Autism Spectrum Disorder (ASD). (**A**) Graph showing Pearson correlation coefficient (r) between challenging behaviours in the cohort. (**B**) Proposed model showing the relationship of challenging behaviour and health status which can influence the severity of the clinical symptoms associated with Autism Spectrum Disorders.

Negative emotional behaviours are commonly observed in individuals with ASD and impact the family and carers around them. When assessing these behaviours in our cohort we found increased irritability and low mood was the most frequently documented behaviour (59.7%) followed by agitation (44.4%), aggression (36.10%), avoidance behaviour (29.2%), tantrums (26.4%), and self-harm (18.1%) (Table 2). As these behaviours are not usually present alone, we further determined if there are any significant correlations between these behaviours (Figure 2). Increased irritability and low mood were found to be significantly correlated with anxiety (r = 0.301; *p* = 0.01) and avoidant behaviour (r = 0.259; *p* = 0.028). When looking at the most significant behavioural correlations for the other negative behaviours, agitation and self-harm both correlated with crying for no reason (r = 0.655; *p* = 0.000) (r = 0.453; *p* = 0.000), aggression and avoidant behaviour both correlated with frequent blinking, sudden screaming, turning in a circle and fixed gaze (r = 0.468; *p* = 0.000) (r = 0.431; *p* = 0.000), and tantrums correlated with facial grimaces (r = 0.403; *p* = 0.000). Further correlations between behaviours are shown in Figure 2.

#### 3.1.3. Changes in Routine and Skills

Routine is important for those with ASD and any deviation from these routines can be difficult for them to manage. When assessing changes in routine, we found changes in appetite or dietary requirement (52.8%) and alterations in sleeping patterns (30.6%), Table 2). As changes in routine can significantly disrupt those with ASD, we conducted a correlation to determine if there is an association between changes in routine and specific challenging behaviours. Changes in appetite correlated significantly with increased irritability and low mood (r = 0.244, *p* = 0.039), and alterations in sleep patterns correlated with avoidant behaviour (r = 0.386, *p* = 0.001). Furthermore, individuals with ASD have difficulty in the acquisition and maintenance of new skills; when assessing this within our cohort 29.1% of respondents reported participants experienced a loss of previously acquired skills. We further looked at which challenging behaviours this loss of skill was associated with and found that it was significantly correlated with tantrums (r = 0.0378; *p* = 0.001). Further correlations between behaviours are shown in Figure 2.

#### 3.1.4. Motor and Vocal Behaviour

Repetitive motor behaviours are a key feature of ASD and were observed in 39% of our cohort. Other motor behaviours that were reported included frequent blinking, sudden screaming, turning in a circle and fixed gaze (38.9%), covering the ears with the hands (37.5%), teeth grinding (bruxism) (34.7%), putting objects in the mouth (30.6%), and facial grimaces (tics) (30%) (Table 2). When looking at which challenging behaviours these motor behaviours had the most significant correlation with, we found that frequent blinking, sudden screaming, turning in a circle and fixed gaze correlated with aggression (r = 0.468, *p* = 0.000), and both covering the ears with the hands and repetitive movements had the highest correlation with one another (r = 0.433; *p* = 0.000). Furthermore, teeth grinding correlated with frequent vocal expressions (r = 0.413; *p* = 0.000), putting objects in the mouth correlated with crying for no reason (r = 0.386; *p* = 0.001), and facial grimaces correlated with tantrums (r = 0.403; *p* = 0.000). In our cohort, frequent vocal expressions were reported in 33.3% of participants and crying for no apparent reason in 31.9% (Table 2). When looking at which behaviours these vocalisations correlate the most with, we found that frequent vocal expressions significantly correlated with both requests for tactility (r = 0.413; *p* = 0.000) and teeth grinding (r = 0.413; *p* = 0.000). Crying for no reason correlated with agitation (r = 0.655; *p* = 0.000). In addition, requests for the tactility of body parts were reported in 26.4% of respondents and were found to correlate with frequent vocal expression most significantly (r = 0.413; *p* = 0.000). Further correlations between behaviours are shown in Figure 2.

### 3.2. The Relationship between Challenging Behavioural Outcomes with Health Status

Next, we sought to determine which behaviours are the most frequent with changes in health. We found that changes to appetite or dietary preferences were the challenging behaviour that had the greatest mean score for its association with a change in health (Table 3, μ = 4.029). Other challenging behaviours that had a high mean score included irritability and low mood (μ = 4.023), loss of previously acquired skills (μ = 3.952), vocal expressions (μ = 3.792), frequent blinking, sudden screaming, turning in a circle and fixed gaze (μ = 3.786), request for tactility (μ = 3.737), and agitation (μ = 3.656). The score for other challenging behaviours can be seen in Table 3.

## 4. Discussion

Autistic children and teenagers may have multiple health concerns and challenging behaviours. However, the relationship between these two distinct clinical phenotypes is poorly understood. This knowledge is important to provide evidence-based treatment approaches by clinicians at specialised service centres and for caregivers to provide support and management strategies for the affected individuals. Furthermore, these behaviours might indicate a new or worsening health problem. To address this knowledge gap, we aimed to determine which challenging behaviours are frequently observed during a change in health in the Macedonian population. Altered appetite or dietary preferences, observed in over half of our participants (52.8%) was one of the significant challenging behaviours associated with changes in health. These findings are in alignment with previously published studies that report selective eating patterns in children with ASD [14]. Although, changes to appetite during illness are not isolated to those with ASD, as most children that experience illness has been shown to have a reduced food intake by 15–20% whilst experiencing upper respiratory infections, diarrhoea, or fevers [19]. However, food selectivity has been shown to occur more frequently in children with ASD compared to those without [20], for which the biological basis largely remains unknown. Some studies have suggested atypical sensory stimulation to be the likely cause towards food selectivity [21,22]. This food selectively in autistic children might impact nutritional adequacy, leading to compromised health of the affected individual [23]. Several studies suggest that children with autism with age is likely to outgrow the tendency of food selectivity [24,25]. It remains to be seen in upcoming studies if this holds true in the Macedonian cohort.

We also found that the loss of previously acquired skills was one of the most significantly associated behaviours with a change in health status. Published studies have reported that a loss of skill, or developmental regression, occurs in around one-third of young children with ASD [26,27,28]. While others do not find any developmental regression in autistic children [29,30]. To our knowledge, our study provides, for the first time, evidence demonstrating that the loss of skills is attributed to a change in health. Although we cannot conclude that a change in health is the cause of this loss of acquired skill, we can speculate that this might have arisen due to cortical malformation and can be associated with the excitatory inhibitory balance of cortical neurons [31,32]. It is likely that health conditions have negative impacts on functional cortical connectivity, which has been shown to be comprised in subjects with ASD [33,34]. We propose that the physiological effects of a health condition may be the catalyst that causes the regression seen in those with ASD. Although, due to the nature of this study, we were unable to conclude which types of health conditions were associated with the loss of a previously acquired skill. Therefore, this warrants further research into the specific changes in health that are associated with challenging behaviours in ASD.

As it is difficult for those with ASD to communicate with carers/parents, treatment of illness or other health conditions unrelated to ASD may be delayed. By demonstrating in our study that changes in appetite or dietary preference, increased irritability and low mood, loss of previously acquired skills, and vocal expression were associated with a change in health, we propose these behaviours can be used by parents/carers as an early indicator that the individual with ASD may be experiencing illness and to be vigilant for any changes in health to discuss it medical professionals for early intervention. Although, due to the subjective nature and variability between behaviours, it is difficult to assume the behaviours stated above apply to all those with ASD. Therefore, the use of behavioural changes to prompt medical-seeking behaviour should not be used as an isolated way of determining changes in health, but in conjunction with other accessible ways of determining signs of illness.

The questionnaire distributed to parents/carers was formed of challenging behaviours that were those suggested in a study by Biue et al. [7]. Although the behaviours suggested in the above study were for abdominal pain, we felt these suggested behaviours may manifest in a wider range of health conditions. The design of this study has an advantage in that it allows us to look at a broad range of health problems and behaviours, thus increasing our chances of finding associations between them. It also has the advantage of the direct caregiver of the subject, who can accurately report on the behaviour of the subject. However, it also has limitations; if the symptoms were not suspected of the behaviours, then no investigation may have been done to look for health problems, resulting in the under-reporting of symptoms associated with a change in health. Furthermore, our study design was limited by the subjective nature of behavioural observations made by the parents/carers and did not provide us with in-depth detail regarding the severity of changes in health. We propose that a functional behavioural assessment could be conducted during a change in health to further understand these observed difficult behaviours.

Although the parents and caregivers are the ones who are more familiar with challenging behaviour, parenting self-efficacy could have influenced the outcome of this study. This could primarily be due to a lack of communication deficits in autistic children and parents. It has been demonstrated that, in comparison to paternal parents, mothers of young autistic children are likely to get more stressed [35,36]. This suggests that scores given by parents or caregivers to questioners are likely to have been influenced by gender and the understanding of questions by caregivers. Furthermore, some studies have suggested that parents of borderline or subthreshold autism are more stressed in comparison to parents of children with severe autism [37], suggesting that the clinical phenotype of children might also have influenced the score given by the parents. We acknowledge that the non-inclusion of the control group is a limitation of our study, and we did not include it as we wanted to first establish the behavioural phenotype in autistic individuals.

In this study, we looked at the correlation between different types of challenging behaviours and health conditions, but we did not have enough information to establish the association between health conditions and changes in behaviour. However, our study design had limitations; it was not able to establish an association with types of health conditions and manifestation of challenging behaviour. In future questionnaires, we suggest that questions regarding the types of health conditions that are associated with challenging behaviours be included, which would also help to validate the working model we have proposed (Figure 2B). This will allow us to determine which types of health conditions are associated with difficult behaviours in autism spectrum disorders. We propose that functional behavioural assessment could be conducted during hospital admission or GP consultation, as well as collecting the relevant clinical information of the admission or consultation. Future prospective studies will give better insight if these calling behaviour regressed or compounded with age and if these are associated with health status.

## 5. Conclusions

Our study provides early evidence that changes in health are important influences of challenging behaviours in subjects with Autism Spectrum Disorder in the Macedonian population. We found that appetite or dietary preferences, irritability and low mood, and loss of previously acquired skills were associated with changes in health. Based on this we recommend that caregivers and family members should look out for the distress behaviours for better management to improve the psychological wellbeing of the person with ASD. The future direction of this work is on the physiological causation of these observed challenging behaviours arising from a change in health.

## Figures and Tables

**Table 1:** Demographic data.

	Min	Max	Mean	Std. Deviation
Age	3	24	10.53	4.07
Height	76	180	142.60	26.06
Weight	15	95	46.26	20.15
Volume in half	23	128	63.46	20.13

**Table 2:** Challenging behaviours.

	♂ N	♀ N	%	Mean	Std. Deviation	Std. Error Mean
Loss of previously acquired skills	19	2	29.1	3.95	0.740	0.161
Irritability and low mood	34	10	59.7	4.02	0.963	0.147
Tantrums and oppositional behaviour	15	4	26.4	3.37	10.257	0.288
Frequent night waking or general sleep disturbance	17	7	30.6	3.50	10.142	0.233
Change in appetite or dietary preferences	28	10	52.8	4.08	10.024	0.166
Heightened anxiety and/or avoidance behaviours	25	12	52.8	3.70	10.077	0.177
Repetitive rocking or other new repetitive movement	19	9	39	3.18	10.362	0.257
Covering ears with hands	21	7	37.5	3.18	10.278	0.242
Teeth grinding	18	7	34.7	3.64	10.254	0.251
Posturing or seeking pressure to specific area	17	2	26.4	3.74	10.195	0.274
Behaviour around evacuation	14	7	29,2	3.62	10.117	0.244
Aggression: onset of, or increase in, aggressive behaviour	20	6	36.1	3.65	10.164	0.228
Self-injurious behaviour: biting, hitting/slapping face, head-banging, unexplained increase in self-injury	11	2	18.1	3.54	10.330	0.369
Facial grimacing or brow furrowing, wincing, tics	19	5	30	3.08	10.349	0.275
Mouthing behaviours: chewing on clothes	18	4	30.6	3.09	0.921	0.196
Sobbing ‘for no reason at all’	15	8	31.9	3.61	0.988	0.206
Vocal expressions: moaning, groaning, sighing, whining	18	6	33.3	3.79	0.977	0.199
Agitation: pacing, jumping up and down	23	9	44.4	3.66	10.035	0.183
Blinking, sudden screaming, spinning and fixed look	20	8	38	3.79	10.101	0.208

♂ = Male; ♀ = Female.

**Table 3:** Challenging behaviour and association with changes in health status.

	95% Confidence Interval of the Difference					
	t	df	Sig (2-Tailed)	Mean. Diff.	Lower Score	Upper Score
Loss of previously acquired skills	24.475	20	0.0001	3.952	3.62	4.29
Irritability and low mood	27.386	42	0.0001	4.023	3.73	4.32
Tantrums and oppositional behaviour	11.685	18	0.0001	3.368	2.76	3.97
Frequent night waking or general sleep disturbance	15.013	23	0.0001	3.500	3.02	3.98
Change to appetite or dietary preferences	24.566	37	0.0001	4.079	3.74	4.42
Heightened anxiety and/or avoidance behaviours	20.919	36	0.0001	3.703	3.34	4.06
Repetitive rocking or other new repetitive movement	12.346	27	0.0001	3.179	2.65	3.71
Sensory hyper responsivity: hyperacusis, tactile defensiveness, sensitivity to light	14.062	22	0.0001	3.522	3.00	4.04
Covering ears with hands	13.159	27	0.0001	3.179	2.68	3.67
Teeth grinding	14.510	24	0.0001	3.640	3.12	4.16
Posturing or seeking pressure to specific area	13.636	18	0.0001	3.737	3.16	4.31
Behaviour around evacuation	14.848	20	0.0001	3.619	3.11	4.13
Aggression: onset of, or increase in, aggressive behaviour	16.003	25	0.0001	3.654	3.18	4.12
Self-injurious behaviour: biting, hitting/slapping face, head-banging, unexplained increase in self-injury	9.592	12	0.0001	3.538	2.73	4.34
Facial grimacing or brow furrowing, wincing, tics	11.200	23	0.0001	3.083	2.51	3.65
Mouthing behaviours: chewing on clothes	15.739	21	0.0001	3.091	2.68	3.50
Sobbing ‘for no reason at all’	17.516	22	0.0001	3.609	3.18	4.04
Vocal expressions: moaning, groaning, sighing, whining	19.011	23	0.0001	3.792	3.38	4.20
Agitation: pacing, jumping up and down	19.980	31	0.0001	3.656	3.28	4.03
Blinking, sudden screaming, spinning, and fixed look	18.199	27	0.0001	3.786	3.36	4.21

t—two tailed *t*-test: df; degrees of freedom: Sig: Significance level.

## Data Availability

The data that support the findings of this study are available from the corresponding author upon reasonable request.
